# Lifestyle's influence on community-dwelling older adults' health: A mixed-methods study design

**DOI:** 10.1016/j.conctc.2020.100687

**Published:** 2020-12-13

**Authors:** Catharina Gillsjö, Sandra Karlsson, Fredrik Ståhl, Irene Eriksson

**Affiliations:** a School of Health Sciences, University of Skövde, Skövde, Sweden; b Faculty of Caring Science, Work Life and Social Welfare, University of Borås, Borås, Sweden; c College of Nursing, University of Rhode Island, Kingston, USA; d Department of Natural Science and Biomedicine, School of Health and Welfare, Jönköping University, Jönköping, Sweden

## Abstract

Background: Aging often involves health problems and difficulties, such as physical and psychological impairments, isolation, and loneliness, causing social and existential consequences. Studies have explored aging from different perspectives. However, few studies have examined healthy older adults’ genetic backgrounds, lifestyles, and meaning in life separately or in combination. This study aims to describe how healthy older adults experience aging, health, lifestyles, and meaning in life and explore potential genetic correlations.

Methods and Design:The project will comprise three main parts: a quantitative section featuring the development and testing of a lifestyle questionnaire, a quantitative genetic analysis, and a qualitative interview study. Participants will be community-dwelling, healthy, older adults between 70 and 95 years of age. A sample size of 800 older adults will be invited to participate in seminars in collaboration with the national Swedish association Active Seniors. Data will be collected through lifestyle questionnaire, DNA extracted from saliva samples, and interviews. Based on questionnaire responses, profile groups will be created and compared statistically with variations in genetic backgrounds, providing the basis for recruiting participants to the qualitative interviews.

Discussion: This study’s expected outcome will be to gain knowledge about variations in genetic backgrounds correlated with individual experiences regarding aging, health, and meaning in life. This knowledge can improve the understanding of motivations for healthy lifestyle changes. The results can reveal potential implications for individual prerequisites to healthy aging and how health-promoting aging and lifestyle counseling can be adjusted to meet individual needs.

## 1 Introduction

Worldwide, both the number and the proportion of older adults in the population are rapidly increasing. The number of older adults aged 65 and over is expected to almost double and to constitute nearly 30% of Europe's total population in 2050 [1,2]. The process of aging varies between individuals and is associated with a variety of health problems that often influence daily life. Sometimes, when health problems occur in older adults, healthcare or social service provisions are needed at home or in institutions, which presents a challenge of increased demands and costs for society at large.

Aging as a phenomenon has been explored using different aspects of environmental factors, lifestyles, and genetics. However, studies examining a combination of healthy older adults’ genetic backgrounds, lifestyles, and meaning in life remains limited—although research indicates that various lifestyle factors modify genetic effects on health-related factors, such as body mass index (BMI) [3]. The terms “healthy older adults” and “older adults in good health” are used in this study even to describe older adults who may not be entirely free of disease or undiagnosed health problems. This choice is based on the notion that good health both has an objective and a subjective dimension.

## 2 Background

### 2.1 Genetic background and aging

With the accelerating development of genetic analysis during the last decade significant interest has focused on identifying the specific genes responsible for the aging process. In particular, the so-called genome-wide association studies (GWAS) have made linking specific genes and gene markers to “incidence” of high age possible by comparing gene patterns for very old people (100 years old or more, so-called centenarians) with younger population groups [4].

The genetic influence on aging is substantial, starting at the cellular level. Cells have different means to repair the damage that contributes to aging, and even individual genes have been found to have a significant impact. One example of a specific gene that has attracted considerable attention in recent years is the Target of Rapamycin (TOR). This gene intervenes in many regulatory systems, and it also affects the appearance of several serious diseases (such as cancer, type II diabetes, and cardiovascular disease) [5]. Strangely, in animal models, the inactivation of TOR during the second half of life has been shown to lead to both prolonged life and reduced incidence of many diseases that normally begin in later life stages [6]. Another example is Forkhead Box O3a (FOXO3a), which has been linked to human longevity in several population studies [[7], [8], [9]]. FOXO3a is part of the insulin/insulin-like growth factor (IGF-1) signaling (IIS) pathway, which includes other genes shown to be associated with longevity [4]. In addition, various other genes have been associated with high age, such as raised serum thyroid-stimulating hormone (TSH) [10], apolipoprotein E (APOE), angiotensin-converting enzyme (ACE) [11], and genes included in the telomerase-activating pathway [4].

The aging process is also affected by diet and lifestyle, which affect the amount of damage or repair that occurs in cells and tissues [12]. For example, a prolonged, significantly reduced calorie intake has been shown to have a life-prolonging effect in a wide variety of species—from yeast and roundworms (Caenorobis elegans) to fruit flies (*Drosophila melanogaster*), mice, rats, and even rhesus monkeys [13].

### 2.2 Health, lifestyle, and meaningful aging

Older adults are a vulnerable population, and growing old often involves health problems and difficulties, leading to physical and psychological impairments, isolation, and loneliness. Ultimately, this process may cause social and existential consequences, such as a reduced sense of meaning in life [2,[14], [15], [16]]. These consequences may lead to a passive lifestyle that can, in turn, become a vicious circle affecting physical and psychological health, including quality of life [[17], [18], [19], [20], [21]]. A meaningful life has been associated with a sense of health and wellbeing, as well as increased physical activity [15,22,23], and this association must be recognized and further explored—both in the provision of healthcare and social services and in research.

Older adults' lifestyles and genetic backgrounds affect both longevity and health, but in very old age, lifestyle choices play a minor role compared to the impact of genetic background [24]. However, lifestyle selection is important for older adults, as physical activities' well-documented impact on health clearly exemplifies. For example, estimates have suggested that regular exercise can prolong life by decreasing the risk of developing lifestyle-related diseases [18]. According to Hillman, Erickson, and Kramer [25], a growing number of studies have supported physical exercise's role as a lifestyle factor that can increase physical and psychological health throughout life.

The literature, however, still lacks research oriented toward older adults’ description of their lifestyles, how they perceive good health and well-being, and the factors that influence their lifestyles, perceptions, and meaning in life [cf. 26, 27]. Combining genetic heterogeneity and differences in health with lifestyle, emotional states, and meaning in life may offer clues about how to live a long life in good health, as this study will address.

## 3 Methods

### 3.1 Aim

This study's overall aim is to describe how older adults in good health experience their aging process, health, lifestyles, and meaning in life and to explore potential correlations with genetic backgrounds.

The specific aims of this study are to:

1. Develop and test a questionnaire, comprising a variety of topics in older adults' (aged 70–95 years) earlier and current lives and lifestyles, and collect genetic data through saliva samples
2. Create profile groups based on questionnaire responses and explore correlations with genetic backgrounds
3. Describe individual experiences of health, meaning in life, lifestyles, and healthy aging while understanding the aspects that motivate healthy lifestyle changes, based on the defined profile groups

### 3.2 Design

This project will comprise three main parts to address its specific aims: i) developing and testing a questionnaire addressing a variety of lifestyle factors (Specific Aim 1), ii) comparing gene and lifestyle data (Specific Aim 2), and iii) analyzing a qualitative interview study (Specific Aim 3). The questionnaire will comprise various parts, some of which will be developed within this study and some of which will derive from already-validated instruments regarding aging's influences from lifestyles, emotional states, and meaning in life. The parts developed by the researchers of the current study will comprise variables that describe participants' self-evaluation of health, medication, lifestyles, family and social contacts, and lives before and after retirement.

Participants will be divided into different profile groups, based on similarities in their responses to the questionnaire. These profile groups will be compared statistically with participants’ variations in genetic background, measured by Single Nucleotide Polymorphism (SNP) and methylation microarraying. Profile groups drawn from the responses to the questionnaire will provide the basis for recruiting participants to the qualitative interviews.

### 3.3 Participants

Community-dwelling participants will be recruited in collaboration with the association Active Seniors—a nationwide independent political association in Sweden for older adults above 65 years of age. The participants in this study will be between 70 and 95 years of age and will be living in communities in the southern mid-section of Sweden. They will be informed of the study and invited to participate through advertisements in local newspapers promoting seminars open to everyone in the age group defined above. The seminars will begin with a presentation of the theme “genes and healthy aging.” All participants will have to provide written informed consent before joining the study.

#### 3.3.1 Sample size

To reach an effective power of 80% when simultaneously testing a vast number of individual SNP markers (about 700,000 in this study), the Bonferroni correction generally becomes too stringent since many SNP markers covariate (i.e., identify similar genetic variants). According to Tseng and Young [28], a sample size of about 400 is sufficient when estimated from approximately 700,000 SNP markers. Furthermore, by slightly increasing the effective size of the measured item (the lowest acceptable detected difference in group means), the need for simultaneous tests has been shown to rapidly decrease with a higher number of tests [29]. In this study, this need would correspond to an effect size factor of 2.2 in order to maintain 80% power with a sample size of 800.

Taken together, 800 became the preferred sample size in this study. In addition, by selecting SNP markers known to be associated with specific phenotypes or disorders, the number of simultaneous statistical tests would decrease drastically to numbers in the 1000 or even 100 range. This decrease would, naturally, further increase the statistical power.

### 3.4 Data collection

This study's data collection will be conducted at two different occasions. The first occasion will be a seminar at which specific aims 1 and 2 are addressed through data collection via a questionnaire and saliva samples. The second occasion will be face-to-face interviews that address Specific Aim 3.

#### 3.4.1 Genetic background

Deoxyribonucleic acid (DNA) will be obtained through the collection of saliva samples at gatherings after a presentation about the aging process. Participants will be informed not to eat or drink, other than drinking water, during the 45-min presentation in order to avoid contamination of the saliva samples. Instructions for data collection will be provided after the presentation. The samples will be marked with participants’ names and years of birth, and the extracted DNA will be subjected to DNA-marker analysis using microarray techniques.

#### 3.4.2 Questionnaire

The questionnaire will include items regarding demographic data, medical history, family relationships (family, children, and grandchildren), and self-evaluations of health, lifestyle, and meaning in life. To quantitatively evaluate participants' sense of coherence, emotional states, and meaning in life, three validated protocols will be included in this study's questionnaire: Sense of Coherence – 13 (SOC-13), Sense of Coherence-Emotional (SOC-E), and Sense of Meaning Profile (SOMP) [[30], [31], [32], [33], [34]]. Participants will be requested to answer open-ended questions about their actual life situations, lifestyles, and changes in lifestyle since retirement. The questionnaire's layout and formulation will be tested by recruiting a subset of approximately 20 former alumni with spouses whose comments will be recorded via face-to-face interviews.

#### 3.4.3 Interviews

A lifeworld approach will be used in the interviews, focusing on the world as it is experienced [35]. In total, 30 to 40 qualitative interviews will be conducted to gain a deeper understanding of older adults' health, meaning in life, lifestyles, and healthy aging and to understand the aspects that motivate healthy lifestyle changes. Participants will be selected through a strategic sample drawn from the profile groups to achieve a wide variation in background and experiences. Participants’ responses to the SOC-13, SOC-E, and SOMP questionnaires [[30], [31], [32], [33], [34]], gender, and age will provide the basis for strategic sampling in order to achieve variety. The sampling will focus on variety in combinations of high and low scoring in the questionnaire. Participants will be asked to describe their experiences of health and the changes they have made in their lives, as well as lifestyle changes since retirement, to enhance their health. Furthermore, they will be asked to describe what they experienced as meaningful in life. Participants will be encouraged to reflect and talk openly about their experiences. The interviews will be audio-recorded and transcribed verbatim.

### 3.5 Analysis of data

The collected data will be analyzed using quantitative and qualitative methods. Each participant will be assigned a code that will remain the same throughout the data analysis.

#### 3.5.1 Quantitative data analysis

DNA will be extracted from the collected saliva samples using the PSP SalivaGene DNA Kit (Stratagene). The DNA samples will be sent to the Swegene Centre for Integrative Biology at Lund University (SCIBLU) to array two sets of different DNA markers (the Infinium OmniExpress-24 and the Illumina methylation 450 k kits), encompassing approximately 710,000 and 450,000 markers, respectively. The arrays will be stored in a locked freezer before transportation to the SCIBLU facility. The remaining DNA samples will be stored in a locked freezer. The samples will be analyzed using two different software tools, Genome Studio (Illumina) and R Studio (open-source) to map participants’ DNA-sample distribution. Preprocessing and subsequent analyses of the SNP microarray data will be performed using the R/Bioconductor platform, for example using the CRLMM algorithm or GWAS tools, to produce genotype calls, confidence scores and other quality metrics and both the SNP and sample levels. For analyses of the Infinium DNA microarrays, the Bioconductor package Minfi will be used. Cluster analyses such as K means or hierarchical clustering of the data will also be performed to group the samples according to similar profiles. The questionnaire will be subjected to factor analysis and/or Principal Component Analysis (PCA). The samples will be compared to questionnaire responses using standard statistical methods within the analysis software mentioned above, as well as the IBM SPSS statistical platform (IBM Analytics).

#### 3.5.2 Qualitative data analysis

The interview data will be analyzed using a variety of qualitative methods in order to reach a comprehensive understanding of health, meaning in life, lifestyle, and healthy aging as experienced by older adults and to understand the aspects that motivate healthy lifestyle changes. Example data analysis methods for this study are phenomenology [35], phenomenography [36,37], and hermeneutics [38,39].

### 3.6 Ethical considerations

The principles outlined in the *Declaration of Helsinki* [40] will be followed, and the Regional Ethical Review Board in Gothenburg (989–13) has approved the study. Participants will be informed, both verbally and in writing, and asked to provide their informed consent. They will also be informed that they can decide not to continue at any time, without explanation or negative consequences.

## 4 Discussion of expected outcome

The study's overall expected outcome is to gain knowledge about variations in genetic backgrounds and individual experiences regarding the aging process, health, meaning in life, and healthy aging and to understand the aspects that motivate healthy lifestyle changes. An additional expected outcome is to gain knowledge regarding the prerequisites for healthy aging on an individual basis and how health-promoting lifestyle counseling during the aging process can be adjusted to meet individual needs. If the analysis reveals that each genetic profile is, in some way, associated with responses to items in the questionnaire regarding such variables as health and lifestyle, it may reveal that an individual's choice of lifestyle can undergird and complement certain prerequisites regarding genetic heritage.

Furthermore, a deepened understanding of health, lifestyles, and meaning in life will be achieved through the interviews with participants from the clustered profile groups. In contrast to qualitative studies generally, the researchers in the current study will have the potential to read each participant's questionnaire and, based on this reading, develop an understanding of each participant's background regarding health, lifestyle, social engagements, and meaning in life ahead of the interview process. This approach might, of course, affect the researchers' understanding—but it might also enable a new dimension and breadth in the interviews themselves and in the resulting analysis.

The idea that guides this whole research project is: Whatever genetic condition older adults carry in advanced age, the importance of health status, lifestyle, and lived experience of wellbeing will be evident. Unraveling fortunate combinations of genetic background and lifestyle, wellbeing, and perceived meaning in life may guide other older adults with similar circumstances and conditions.

## Funding

The study is funded by the School of Health and Education, University of Skövde, Sweden, and University of Borås, Academy of Care, Working Life and Social Welfare, Sweden.

## Conflicts of interest

The authors declare that they have no competing interest.

## Authors' contributions

The authors (CG, SK, FS, IE) made substantial contributions in designing the study and prepared the manuscript for submission. All authors have read and approved the final version of the manuscript to be published and agree to be accountable for all aspects of the work related to ensuring accuracy.
